# Exploring the involvement of ferroptosis-associated genes and pathways in mesenchymal stem cell aging through bioinformatics analysis

**DOI:** 10.3389/fragi.2025.1509267

**Published:** 2025-10-15

**Authors:** Laleh Mavaddatiyan, Yasaman Khamineh, Leila Taghiyar, Mahmood Talkhabi

**Affiliations:** ^1^ Department of Animal Sciences and Marine Biology, Faculty of Life Sciences and Biotechnology, Shahid Beheshti University, Tehran, Iran; ^2^ Department of Stem Cells and Developmental Biology, Cell Science Research Centre, Royan Institute for Stem Cell Biology and Technology, ACECR, Tehran, Iran

**Keywords:** mesenchymal stem cells, ferroptosis, aging, enrichment analysis, bioinformatics

## Abstract

Mesenchymal stem cells (MSCs) exhibit self-renewal and multipotent differentiation capabilities, and play roles in tissue repair and regeneration. However, age-related alterations can impair MSCs functions, potentially contributing to accelerated aging processes. Ferroptosis, a regulated form of cell death involving iron-mediated lipid peroxidation, is implicated in age-related diseases, although its specific role in MSCs aging remains unclear. Herein, the GSE68374 dataset was analyzed to obtain ferroptosis-related differentially expressed genes (FRDEGs). Gene Ontology (GO) and Kyoto Encyclopedia of Genes and Genomes (KEGG) pathway analyses were performed, and potential drugs targeting FRDEGs were predicted. Protein-protein interaction (PPI) analysis was conducted and hub genes were identified, which were validated using two independent datasets. Furthermore, an integrated regulatory network of kinases, transcription factors (TFs), and microRNAs was constructed. A total of 131 FRDEGs were screened, which were involved in cellular responses to starvation, oxidative stress, lipid metabolism, cellular senescence, ferroptosis, and cancer pathways. Among twenty hub genes, eight key FRDEGs, including activating transcription factor 3 (ATF3), Enhancer of zeste homolog 2 (EZH2), synuclein alpha (SNCA), prostaglandin-endoperoxide synthase 2 (PTGS2), NADPH oxidase 4 (NOX4), cyclin-dependent kinase inhibitor 2A (CDKN2A), sequestosome 1 (SQSTM1), and interleukin 6 (IL6), were similarly regulated across external datasets, and the expression of these genes was also confirmed by qRT-PCR. These findings highlight the pivotal role of these genes in MSCs aging and ferroptosis, suggesting that targeting them could enhance MSCs regenerative capacity and mitigate the progression of aging-related alterations in MSCs.

## Introduction

Mesenchymal stem cells (MSCs) possess self-renewal capabilities and can differentiate into various cell types, crucial for therapeutic applications such as cell therapy (Jimenez-Puerta et al., 2020). Their survival, function, and longevity are essential for tissue maintenance and regeneration due to their immune-regulating and paracrine properties (Krampera and Le Blanc, 2021). However, several factors can have a negative effect on the function and therapeutic properties of these cells, such as aging (Ahmadi and Rezaie, 2021). Aging is a biological process that is associated with a progressive and irreversible decline in the function of body cells, including MSCs (Kamal et al., 2020; Duong et al., 2022). The loss of MSCs number or function due to aging significantly affects the body’s regenerative capacity (Choudhery, 2021). Studies demonstrate that age-related changes lead to diminished beneficial functions in MSCs secretion and promote negative activities, accelerating the aging process and contributing to age-related diseases (Liu et al., 2020). Therefore, understanding the basic molecular pathways associated with aging that led to the impairment and loss of function of MSCs is valuable for improving their function and developing regenerative medicine.

Ferroptosis is a form of non-apoptotic programmed cell death driven by iron (Fe^2+^)-dependent lipid peroxidation (Liu et al., 2022; Schreiner and Schreiner, 2023). This process begins with the peroxidation of phospholipids, particularly those containing polyunsaturated fatty acids (PL-PUFAs) (Endale et al., 2023), which are susceptible to reactive oxygen species (ROS) (Wang F. et al., 2023). Elevated levels of Fe^2+^ in the cytosol can react with H_2_O_2_ (Fenton reaction), generating hydroxyl radicals that attack PL-PUFAs, leading to membrane lipid peroxidation and loss of integrity (Wang F. et al., 2023). Ferroptosis is implicated in aging and age-related diseases (Coradduzza et al., 2023), with disruptions in iron homeostasis adversely affecting cellular function (Tian et al., 2022). Limited research has focused on ferroptosis in MSCs and their aging. A study found that Engeletin protects bone marrow mesenchymal stem cells (BMSCs) from ferroptosis via the nuclear factor-related antioxidant factor 2 (Nrf2) pathway, enhancing survival and differentiation (Huang et al., 2023). Additionally, ferroptosis negatively impacts the osteogenic differentiation of BMSCs, linking it to age-related bone diseases like osteoporosis (Chen et al., 2023; Feng et al., 2023). Other studies have highlighted ferroptosis’s role in aging across various cell types and its connection to diseases associated with aging (Xiong et al., 2023; Xu et al., 2022).

The aim of this study was to explore the relationship between aging in MSCs and ferroptosis by identifying key genes and molecular mechanisms involved in this process through bioinformatics analysis. To establish a mechanistic link between MSCs aging and ferroptosis, the identified ferroptosis related differentially expressed genes (FRDEGs) were subjected to comprehensive bioinformatics investigations and validated *in vitro* using qRT-PCR. The results indicated that genes associated with MSCs aging contribute to multiple cellular pathways and functions related to ferroptosis. Overall, our findings provide new insights into potential genes related to aging in MSCs and therapeutic targets, enhancing the current understanding of the mechanisms of ferroptosis in aging MSCs.

## Materials and methods

**FIGURE 1:**
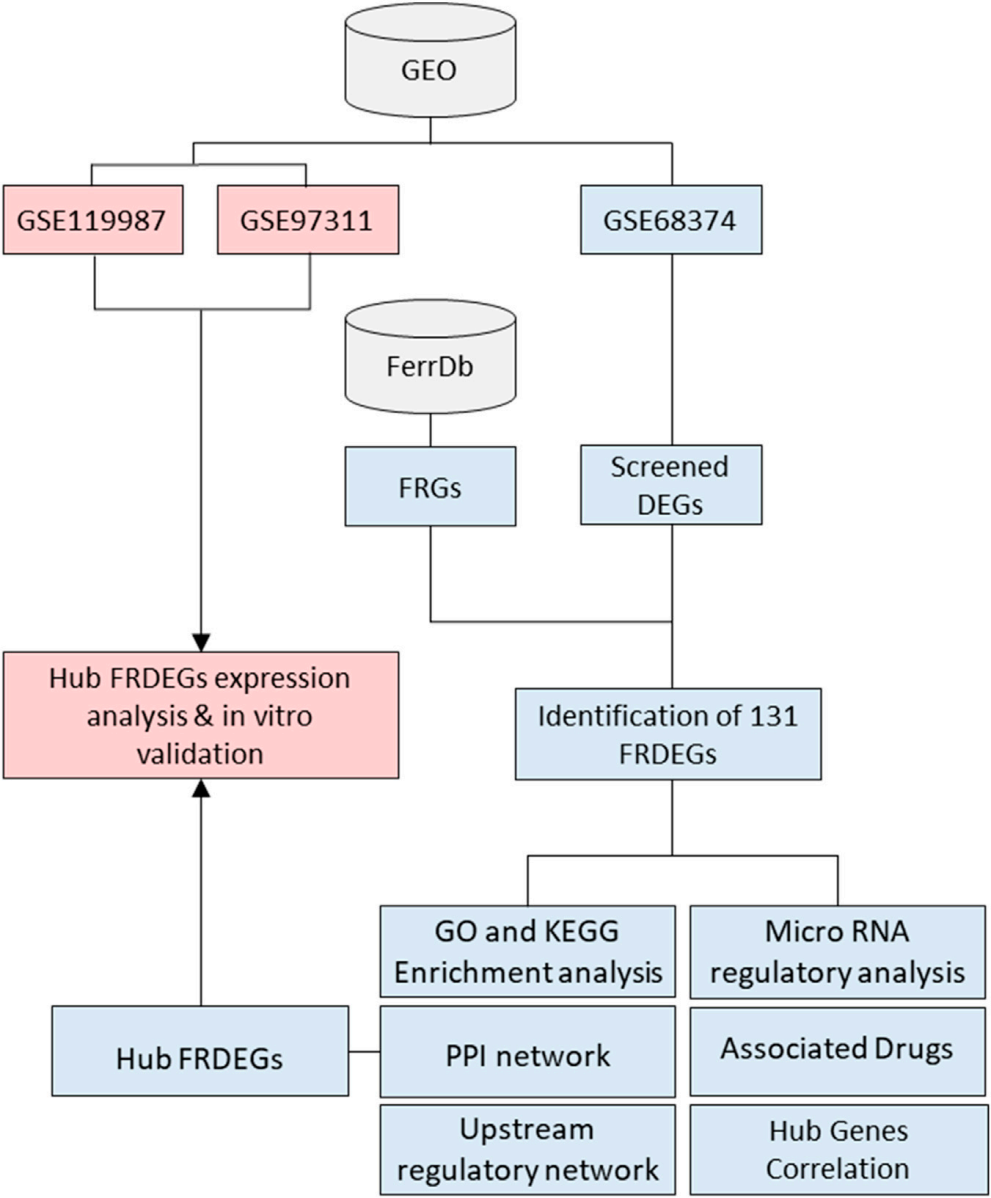
Flowchart illustrating the analysis process from GEO datasets. GSE68374, FRGs, and screened DEGs result in the identification of 131 FRDEGs. Further analyses include GO and KEGG enrichment, PPI network, upstream regulatory network, microRNA regulatory analysis, associated drugs, and hub gene correlation. GSE119987 and GSE97311 lead to hub FRDEGs expression analysis and validation. Origin of the datasets: GSE68374, bone marrow mesenchymal stem cells (BMSCs) derived from both fetal (15–20 weeks) and adult (40–70 years) donors; GSE97311, BMSCs derived from fetal (55 days post conception/7–8 weeks) and aged/adult (60 – 74 years) donors; GSE119987, human umbilical cord MSCs (UC-MSCs) from early (P2) and late (P13-P16) passages. GEO: gene expression omnibus; FRGs: ferroptosis-related genes; DEGs: differentially expressed genes; FRDEGs: ferroptosis-related differentially expressed genes; GO: gene ontology; KEGG: kyoto encyclopedia of genes and genomes; PPI: protein-protein interaction. Blue, indicates the analysis pathway using the original dataset, while pink delineates the validation process using external datasets and *in vitro* validation.

The workflow for the bioinformatics analyses employed to identify FRDEGs in MSCs, analyze the identified FRDEGs, and validate the key hub genes is illustrated in Figure 1.

### Data sources

The study utilized a dataset obtained from the Gene Expression Omnibus (GEO) database (https://www.ncbi.nlm.nih.gov/geo/). The GSE68374 dataset that includes three biological replicates of bone marrow MSCs (BMSCs) derived from both fetal (15–20 weeks) and adult (40–70 years) donors (Paciejewska et al., 2016). Additionally, 472 ferroptosis-related genes, including ferroptosis drivers, ferroptosis suppressors, ferroptosis markers, and unclassified genes, were sourced from the online database FerrDb (http://www.zhounan.org/ferrdb/current/, 10 April 2024).

Additionally, to illustrate the relationship between ferroptosis and aging, aging-related genes obtained from the Aging Atlas (https://ngdc.cncb.ac.cn/aging/index) and Human Ageing Genomic Resources (https://genomics.senescence.info/) databases, as well as genes involved in MSCs aging retrieved from four databases (including DrugBank (https://go.drugbank.com/), GeneCards (https://www.genecards.org/), OMIM (Online Mendelian Inheritance in Man) (https://omim.org/) and PharmGKB (https://www.pharmgkb.org/), were intersected with ferroptosis-associated genes.

### Determination of FRDEGs

The dataset was initially analyzed using gene set enrichment analysis (GSEA) to identify underlying biological pathways and functional mechanisms. Subsequently, the online tool GEO2R and the “limma” package in R were employed to screen for differentially expressed genes (DEGs), with screening criteria set at |log2(FC)| > 1 and P < 0.05. The expression patterns of these DEGs were visualized using heatmaps generated with the “pheatmap” package. To further illustrate gene expression differences, volcano plots were created with the “ggplot2” package in R, providing an intuitive overview of the DEGs. The Venn diagram from the SRplot online tool (https://www.bioinformatics.com.cn/en), was used to identify ferroptosis related differentially expressed genes (FRDEGs) through the intersection of DEGs from the GSE68374 dataset with ferroptosis-related genes obtained from the FerrDb database. Heatmap of FRDEGs was also generated using the “pheatmap” package to facilitate better understanding.

### Functional and pathway enrichment analysis

Functional enrichment analysis of FRDEGs was conducted using Gene Ontology (GO), a globally standardized system for classifying gene functions, which consists of three categories: biological process (BP), cell component (CC), and molecular function (MF). Additionally, the Kyoto Encyclopedia of Genes and Genomes (KEGG) enrichment analysis was utilized to identify relevant signaling pathways. GO and KEGG were performed with “clusterProfiler” package in R (P value < 0.05 was considered statistically significant).

### Protein-protein interaction network construction and identification of hub genes

For PPI network analysis, STRING database (http://string-db.org) was used, which provides experimental and predicted interaction information between two or more proteins. A confidence value of >0.40 was set, and *homo sapiens* was selected as the species. The protein interaction network was constructed, and the STRING analysis results were exported to Cytoscape software version 3.10.1 (https://cytoscape.org/download.html) for visualization. Subsequently, 20 hub genes were identified using the cytoHubba plug-in available in Cytoscape. In order to examine the correlation among hub genes, Pearson’s correlation coefficient was conducted with a significance level of p-value <0.05, and the SRplot online tool was used for visualization.

### Identification of microRNAs regulating FRDEGs

In the present study, three databases, namely, mirDIP (https://ophid.utoronto.ca/mirDIP/), miRWalk (http://mirwalk.umm.uni-heidelberg.de/) and ENCORI (https://rnasysu.com/encori/), were used to predict microRNAs (miRNAs) of FRGEGs. The miRNAs that were present in all three databases for the target genes were considered as core miRNAs.

### Interactions between FRGEGs with kinases and transcription factors

ChEA (https://maayanlab.cloud/Harmonizome/dataset/CHEA) and KEA (https://maayanlab.cloud/Harmonizome/resource/Kinase), found in the Transcription and Pathways sections of Enrichr, respectively, were utilized to identify interactions among transcription factors (TFs), kinases, and FRDEGs. Subsequently, Cytoscape was utilized to visualize the interactions of TFs and kinases with common FRDEGs.

### Identification of drugs associated with FRDEGs

The Drug Signature Database (DSigDB) (http://dsigdb.tanlab.org/), accessible via the Enrichr, was employed to identify drug candidates associated with common FRDEGs. Following the identification of these drugs, potential target genes of them was determined. These target genes were subsequently aligned with FRDEGs, and the intersections were visualized using Cytoscape.

### Validation of the hub FRDEGs

To explore the expression patterns of hub genes, two external datasets were analyzed, GSE97311, which contains BMSCs from fetal (55 days post conception/7–8 weeks) and aged/adult (60–74 years) donors, and GSE119987, which includes human umbilical cord MSCs (UC-MSCs) from early (P2) and late (P13-P16) passages. Initially, both datasets underwent GSEA analysis, followed by the identification of DEGs using the “limma” package in R. The expression patterns of these genes were then investigated in their samples, and finally, they were intersected with genes associated with ferroptosis. Subsequently, the expression changes of the hub FRDEGs in these datasets were validated. Then, Box plots were created to visualize the expression levels of common genes, utilizing the “tidyr,” “ggpubr,” and “rstatix” packages (Saghati et al., 2024) in R software. All scripts and the full R code used for data processing, differential expression analysis, enrichment, and visualization are publicly available in the GitHub repository (https://github.com/laleh-mavaddat/MSCs-aging). P value <0.05 was considered statistically significant.

### Cell culture and expression analysis of validated genes

Human UC-MSCs from a young donor at passage 3 and human BMSCs from an old donor (70 years) at passage 16 were obtained from the Royan Institute. The MSCs were cultured in flasks and maintained at 37 °C in a humidified atmosphere containing 5% CO_2_. To assess the expression levels of validated genes, total RNA was extracted from the cultured cells using the Cell-to-cDNA kit (Hasti Noavaran Gene Royan, Iran), which enables simultaneous RNA extraction and cDNA synthesis. Quantitative real-time PCR (qRT-PCR) was performed using Maxima SYBR Green/ROX qPCR Master Mix (Yekta Tajhiz, Iran) on a StepOnePlus Real-Time PCR System (Applied Biosystems Life Technologies, ABi). Glyceraldehyde-3-phosphate dehydrogenase (GAPDH) served as the housekeeping reference gene for normalization. Relative expression levels of the target gene were calculated using the 2^-ΔΔCt method. The calibrator was the average ΔCt value of the genes in UC-MSC samples (n = 6 biological replicates). The sequences of qRT-PCR primers for the genes examined are listed in the Supplementary Material (Supplementary Table S1). The significance of differences among multiple groups was evaluated using t-test. Statistical significance was defined at a threshold of *p* < 0.05.

## Results

### Identification of FRDEGs

**FIGURE 2:**
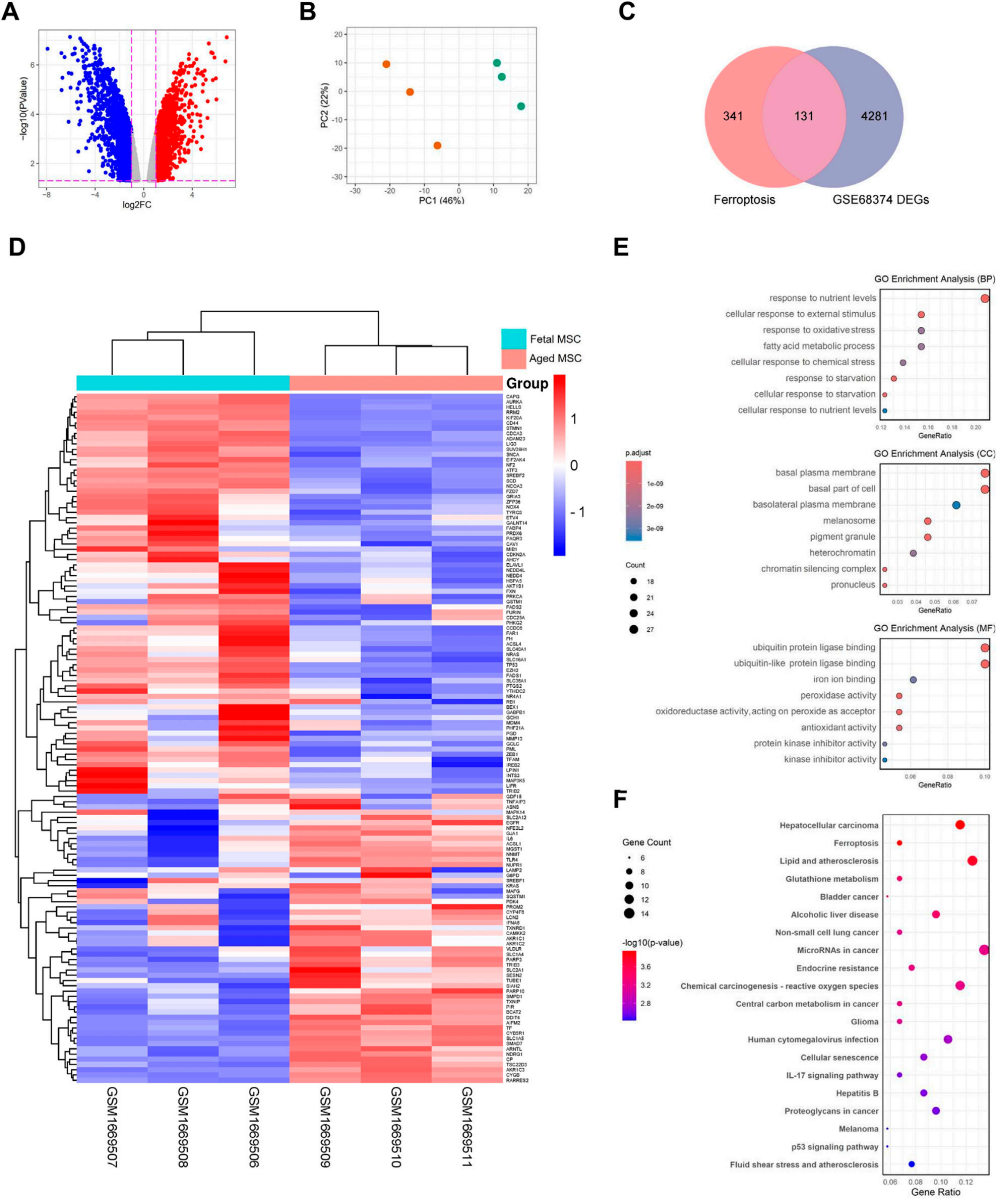
Differential expression analysis of dataset, intersecting FRDEGs and functional enrichment analysis of 131 FRDEGs. **(A)** Volcano plot depicting gene expression changes with points in blue representing downregulated genes and red representing upregulated genes; **(B)** Principal Component Analysis (PCA) plot showing sample groupings; **(C)** Venn diagram showing shared between FRGs and GSE68374 DEGs; **(D)** Heatmap displaying hierarchical clustering of gene expression of 131 FRDEGs across samples from fetal and adult MSCs; **(E)** Dot plots for GO enrichment analysis in Biological Process (BP), Cellular Component (CC), and Molecular Function (MF) categories; **(F)** Dot plot showing KEGG enrichment analysis with gene count and significance levels. FRDEGs: ferroptosis-related differentially expressed genes; FRGs: ferroptosis-related genes; DEGs: differentially expressed genes; FRDEGs: ferroptosis-related differentially expressed genes; GO: gene ontology; KEGG: kyoto encyclopedia of genes and genomes.

The analysis of the intersection between ferroptosis and aging-related genes revealed that out of 472 ferroptosis-associated genes, 54 are involved in aging (Supplementary Materials 1, 2). Heatmaps for the DEGs from GSE68374 showed changes in gene expression between fetal and adult samples and GSEA indicated that the they are primarily involved in pathways such as polyunsaturated fatty acid metabolism, p53 and MAPK signaling pathways, and reactive oxygen species metabolism (Supplementary Figure S1). Subsequently, to investigate the expression patterns of DEGs (Supplementary Material 2), a volcano plot was created (Figure 2A). The plot revealed the relationship between the magnitude of changes in gene expression and the statistical significance of those changes. Additionally, PCA plot demonstrated distinct sample groupings between fetal BMSCs compared to adult BMSCs (Figure 2B). Next, DEGs related with ferroptosis were determined by intersecting the total DEGs of dataset with ferroptosis-related genes. This resulted in the identification of 131 FRDEGs as DEGs related with ferroptosis (Figure 2C, Supplementary Material 2). The hierarchical cluster heatmap illustrated clear differences in the expression of 131 FRDEGs among the study groups (Figure 2D).

### Enrichment analysis of FRDEGs

Enrichment analyses were carried out to determine the function and signaling pathway of FRDEGs. GO analysis for BP showed that FRDEGs are more involved in that cellular response to starvation, cellular response to reactive oxygen species and fatty acid metabolic process. In terms of CC, analysis showed that FRDEGs are mainly enriched in lipid droplet, organelle outer membrane and basolateral plasma membrane. MF analysis revealed significant enrichment of FRDEGs in iron ion binding, antioxidant activity and kinase activity (Figure 2E). Additionally, enrichment analysis of the signaling pathway revealed significant involvement of FRDEGs in cellular senescence, human cytomegalovirus infection, ferroptosis, and pathways in cancer (Figure 2F).

### PPI network analysis of FRDEGs

**FIGURE 3:**
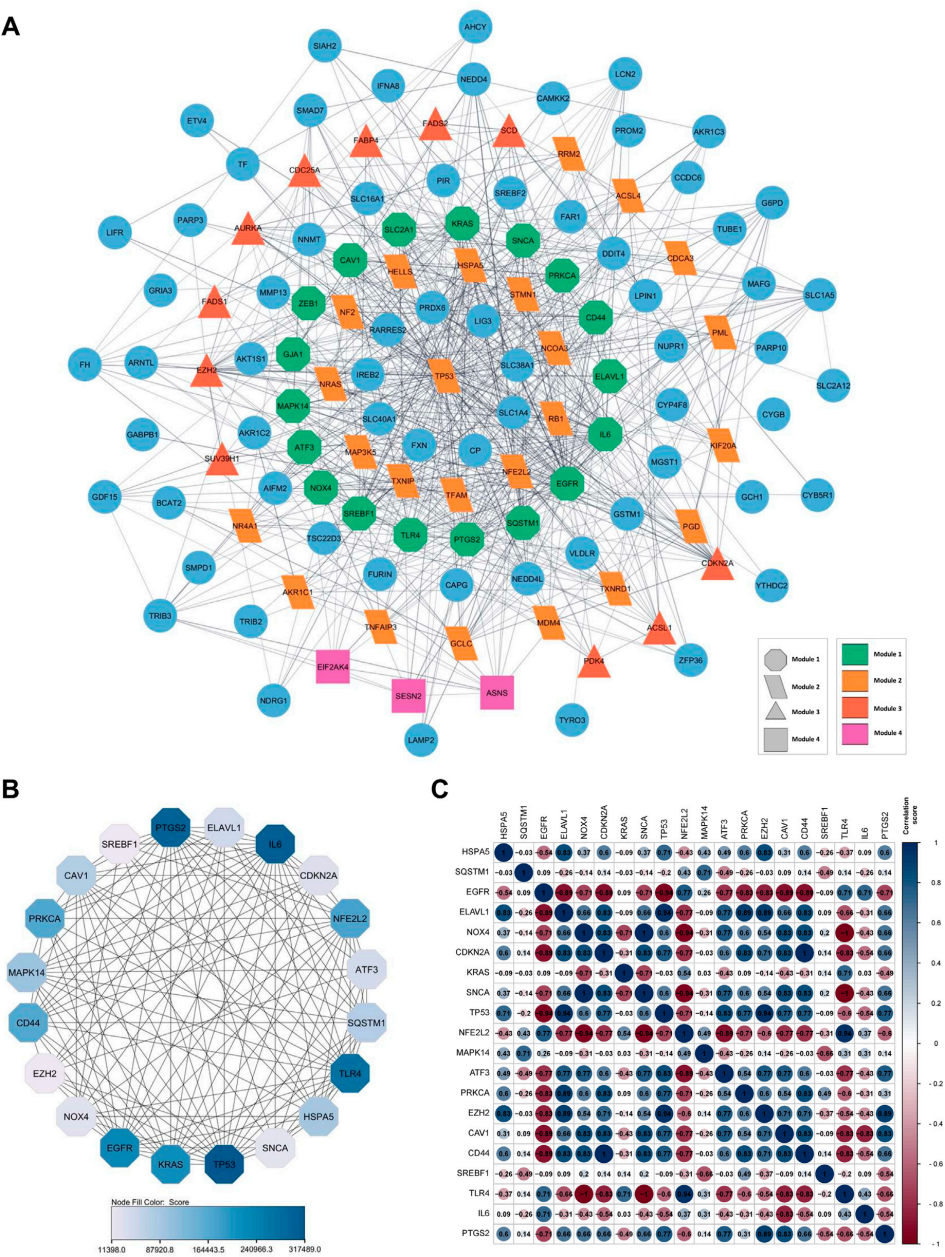
A visual representation of gene or protein interaction networks and data correlation of 131 FRDEGs. **(A)** PPI of FRDEGs shows a multi-colored network with nodes in circles, triangles, rectangles, and other shapes representing different modules connected by lines; **(B)** The PPI network of hub genes; **(C)** Heatmap of hub-genes expression correlation, with circular-colored cells indicating correlation values, accompanied by a color scale bar on the right. Each panel illustrates complex biological interactions. FRDEGs: ferroptosis-related differentially expressed genes; PPI: protein-protein interaction.

The PPI network was constructed to represent the 131 FRDEGs interactions (Figure 3A). It revealed that the FRDEGs comprise a total of 131 nodes and 642 edges. Within the network, genes exhibiting the highest degree of interaction with one another are identified as modules. Based on this, four significant modules were obtained, indicated by distinct shapes and colors (Figure 3A). Module 1, the main module, has 18 genes, while the other modules contain 24, 11, and 3 genes, respectively. Next, 20 FRDEGs were identified as critical genes with the highest number of connections in the PPI network, referred to as hub genes (Figure 3B, Supplementary Material 2). In addition, the hub genes were subjected to correlation analysis, which revealed predominantly positive correlations among them. This suggests that these hub genes collectively exert a synergistic influence on gene expression. Notably, the most strongly correlated genes were TP53 and ELAVL1, NFE2L2 and TLR4, and EZH2 and TP53 (r = 0.94) (Figure 3C).

### MiRNAs regulating FRDEGs

MiRNA analysis revealed that 189 miRNAs target 60 FRDEGs, with some miRNAs targeting multiple genes (Supplementary Table S2). Among these, the main common genes between the datasets (ATF3, EZH2, PTGS2, and SNCA) are targeted by hsa-miR-324-5p, hsa-miR-4640-5p, hsa-miR-4726-5p, hsa-miR-5047, hsa-miR-543, and hsa-miR-3614-5p (Table 1). These miRNAs have the potential to act as biomarkers for ferroptosis and aging.

**TABLE 1:** Main miRNAs targeting validated genes.

miRNAs (mirDIP ∩ miRWalk ∩ ENCORI)	Gene symbol	Gene expression	Ferroptosis driver/Suppressor/Marker/Unclassified
hsa-miR-324-5p hsa-miR-4640-5p hsa-miR-4726-5p	ATF3	Downregulation	Driver/Unclassified
hsa-miR-5047	EZH2	Downregulation	Suppressor
hsa-miR-543	PTGS2	Downregulation	Marker
hsa-miR-3614-5p	SNCA	Downregulation	Driver

### Upstream regulator analysis of FRDEGs

**FIGURE 4:**
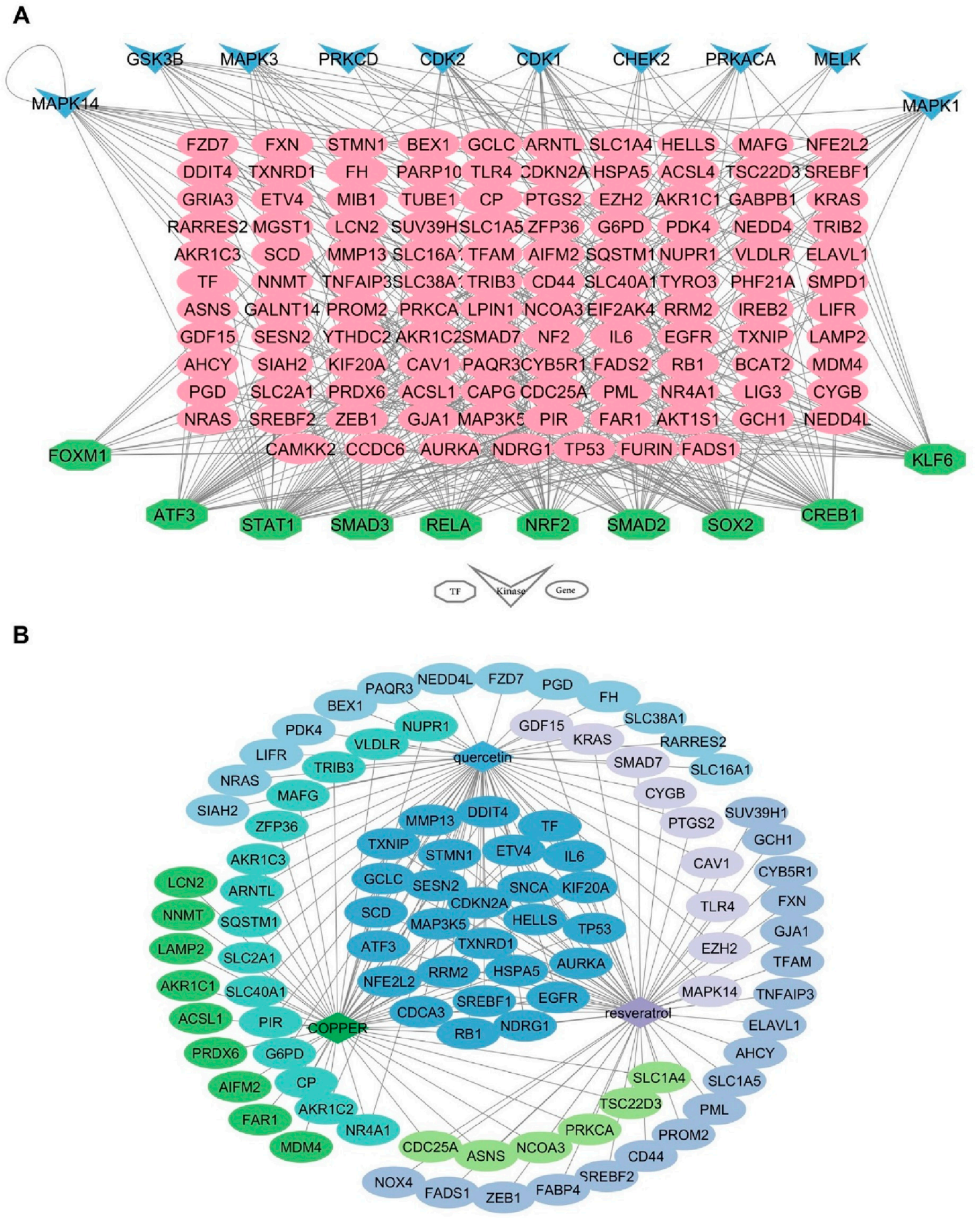
Upstream regulatory and drugs networks of 131 FRDEGs. **(A)** Interaction network of TFs and kinases with FRDEGs; **(B)** The interaction network of drugs and FRDEGs. FRDEGs: ferroptosis-related differentially expressed genes; TFs: transcription factors.

Based on the analysis of TFs and kinases, the top 10 TFs and kinases were identified. Among these, nuclear factor erythroid 2-related factor 2 (NRF2), activating transcription factor 3 (ATF3), signal transducer and activator of transcription 1 (STAT1), and cyclic-AMP response element-binding protein 1 (CREB1) were deemed the most significant transcription factors, while cyclin-dependent kinase 1 (CDK1) and mitogen-activated protein kinase 14 (MAPK14) were recognized as the most important kinases due to their ability to target a greater number of FRDEGs (Figure 4A).

### Drugs associated with FRDEGs

According to drug-related analysis, the top 10 drugs that had the most overlap with the 131 FRDEGs were listed in Table 2. Among these top 10 drugs, copper, resveratrol, and quercetin demonstrated the most interactions with the FRDEGs (Figure 4B).

**TABLE 2:** The top ten potential drugs targeting FRDEGs.

Drugs name	P Value	Genes
COPPER CTD 00005706	3.36E-27	RB1; SLC2A1; SLC40A1; VLDLR; ZFP36; AIFM2; LAMP2; SESN2; STMN1; MAP3K5; SREBF1; HELLS; G6PD; ACSL1; NCOA3; TSC22D3; PRKCA; ETV4; CDC25A; MMP13; DDIT4; TXNIP; FAR1; TRIB3; NUPR1; KIF20A; SQSTM1; TP53; ATF3; NNMT; CDCA3; SLC1A4; NDRG1; EGFR; AURKA; ARNTL; SNCA; RRM2; HSPA5; CDKN2A; TXNRD1; AKR1C1; AKR1C3; ASNS; AKR1C2; PRDX6; CP; NR4A1; GCLC; TF; IL6; SCD; MAFG; LCN2; PIR; MDM4; NFE2L2
resveratrol CTD 00002483	2.46E-31	RB1; SUV39H1; TNFAIP3; ELAVL1; GJA1; SESN2; STMN1; FADS1; MAP3K5; SREBF1; HELLS; NCOA3; TSC22D3; PRKCA; ETV4; CDC25A; SREBF2; MMP13; ZEB1; DDIT4; TFAM; TXNIP; KIF20A; TP53; TLR4; FXN; ATF3; CD44; AHCY; CDCA3; SLC1A4; SLC1A5; PTGS2; NDRG1; EGFR; CYGB; AURKA; CYB5R1; SNCA; PROM2; RRM2; HSPA5; GCH1; GDF15; CDKN2A; CAV1; TXNRD1; ASNS; MAPK14; PML; SMAD7; GCLC; TF; IL6; FABP4; SCD; NOX4; KRAS; EZH2; NFE2L2
quercetin CTD 00006679	7.91E-19	RB1; SLC2A1; SLC40A1; VLDLR; ZFP36; SESN2; STMN1; PDK4; MAP3K5; SREBF1; HELLS; G6PD; SLC38A1; PAQR3; LIFR; PGD; ETV4; MMP13; DDIT4; TXNIP; TRIB3; NUPR1; KIF20A; SQSTM1; TP53; TLR4; ATF3; FH; BEX1; CDCA3; NEDD4L; PTGS2; NDRG1; EGFR; CYGB; AURKA; ARNTL; NRAS; SNCA; SLC16A1; RRM2; HSPA5; GDF15; CDKN2A; RARRES2; FZD7; SIAH2; CAV1; TXNRD1; AKR1C3; AKR1C2; MAPK14; CP; SMAD7; NR4A1; GCLC; TF; IL6; SCD; MAFG; PIR; KRAS; EZH2; NFE2L2
Arsenenous acid CTD 00000922	2.31E-24	RB1; FH; SLC2A1; SLC1A4; VLDLR; PTGS2; EGFR; ZFP36; GJA1; AIFM2; LAMP2; PDK4; MAP3K5; SLC16A1; RRM2; HSPA5; GDF15; ACSL1; CDKN2A; CAV1; TXNRD1; AKR1C1; AKR1C3; AKR1C2; PRKCA; MAPK14; PGD; PRDX6; CDC25A; PML; SMAD7; NR4A1; GCLC; IL6; MMP13; ZEB1; FABP4; MAFG; DDIT4; PIR; TFAM; KRAS; TRIB3; TP53; FXN; TRIB2; ATF3; NFE2L2
Tetradioxin CTD 00006848	2.29E-23	RB1; SLC2A1; SLC40A1; TNFAIP3; VLDLR; ELAVL1; FADS2; ZFP36; GJA1; AIFM2; SMPD1; STMN1; PDK4; FADS1; MAP3K5; SREBF1; HELLS; ACSL1; NCOA3; PAQR3; LIFR; LIG3; PRKCA; CDC25A; MMP13; DDIT4; TFAM; TXNIP; TRIB3; NUPR1; KIF20A; TP53; TRIB2; CD44; CDCA3; MGST1; NEDD4L; CAPG; PTGS2; NDRG1; EGFR; CYGB; AURKA; GRIA3; SLC16A1; GSTM1; RRM2; HSPA5; GDF15
benzo [a]pyrene CTD 00005488	7.06E-22	RB1; GALNT14; SLC2A1; SLC40A1; TNFAIP3; VLDLR; ZFP36; GJA1; AIFM2; SESN2; STMN1; PDK4; MAP3K5; SREBF1; G6PD; SLC38A1; ACSL1; NCOA3; ACSL4; LIFR; PRKCA; ETV4; CDC25A; SREBF2; MMP13; DDIT4; TFAM; TXNIP; FAR1; TRIB3; NUPR1; KIF20A; SQSTM1; TP53; ATF3; CD44; BEX1; CDCA3; MGST1; NEDD4L; FURIN; CAPG; SLC1A5; PTGS2; NDRG1; EGFR; CYGB; AURKA; ARNTL; CYB5R1; ADAM23; GRIA3; SNCA; GSTM1; RRM2; HSPA5; GDF15; RARRES2; FZD7
withaferin A MCF7 UP	7.10E-22	GDF15; TXNRD1; AKR1C1; AKR1C3; ASNS; AKR1C2; SLC1A4; VLDLR; GABPB1; NDRG1; GCLC; MAFG; TRIB3; NUPR1; LPIN1; SQSTM1; ATF3
7646–79–9 CTD 00000928	4.97E-20	SLC2A1; VLDLR; FADS2; GJA1; AIFM2; LAMP2; STMN1; FADS1; SREBF1; G6PD; SLC38A1; NCOA3; PAQR3; ACSL4; PRKCA; PGD; SREBF2; ZEB1; DDIT4; TFAM; TXNIP; TRIB3; KIF20A; SQSTM1; TP53; FXN; ATF3; CD44; BCAT2; FH; NNMT; CDCA3; MGST1; FURIN; SLC1A5; PTGS2; NDRG1; EGFR; PHF21A; AURKA; CAMKK2; CYB5R1; ADAM23; SNCA; RRM2; GDF15; CAV1; AKR1C1; AKR1C3; AKR1C2; CP; PML; GCLC; TF; IL6; FABP4; SCD; MAFG; LCN2; PIR; CCDC6; NOX4; NF2; LPIN1; EZH2
Retinoic acid CTD 00006918	5.18E-20	RB1; GALNT14; SLC2A1; SLC40A1; VLDLR; ELAVL1; GJA1; SMPD1; LAMP2; STMN1; PDK4; MAP3K5; G6PD; SLC38A1; ACSL1; NCOA3; TSC22D3; LIFR; PRKCA; PGD; CDC25A; SREBF2; ZEB1; DDIT4; TYRO3; NUPR1; SQSTM1; TP53; TLR4; TRIB2; ATF3; CD44; BCAT2; FH; BEX1; CDCA3; MGST1; CAPG; PTGS2; NDRG1; EGFR; PHF21A; CYGB; ARNTL; CYB5R1; ADAM23; GRIA3; SNCA; PROM2; SLC16A1; GSTM1; RRM2; HSPA5; GCH1; GDF15; CDKN2A; RARRES2; FZD7; CAV1
troglitazone CTD 00002415	2.00E-19	RB1; SUV39H1; SLC2A1; TNFAIP3; PTGS2; NDRG1; FADS2; ZFP36; SESN2; STMN1; PDK4; MAP3K5; SREBF1; GSTM1; RRM2; HSPA5; GDF15; ASNS; CDC25A; SREBF2; GCLC; IL6; FABP4; DDIT4; TFAM; TRIB3; KIF20A; SQSTM1; TP53; ATF3; EZH2; NFE2L2

### Validation of the FRDEGs hub genes

**FIGURE 5:**
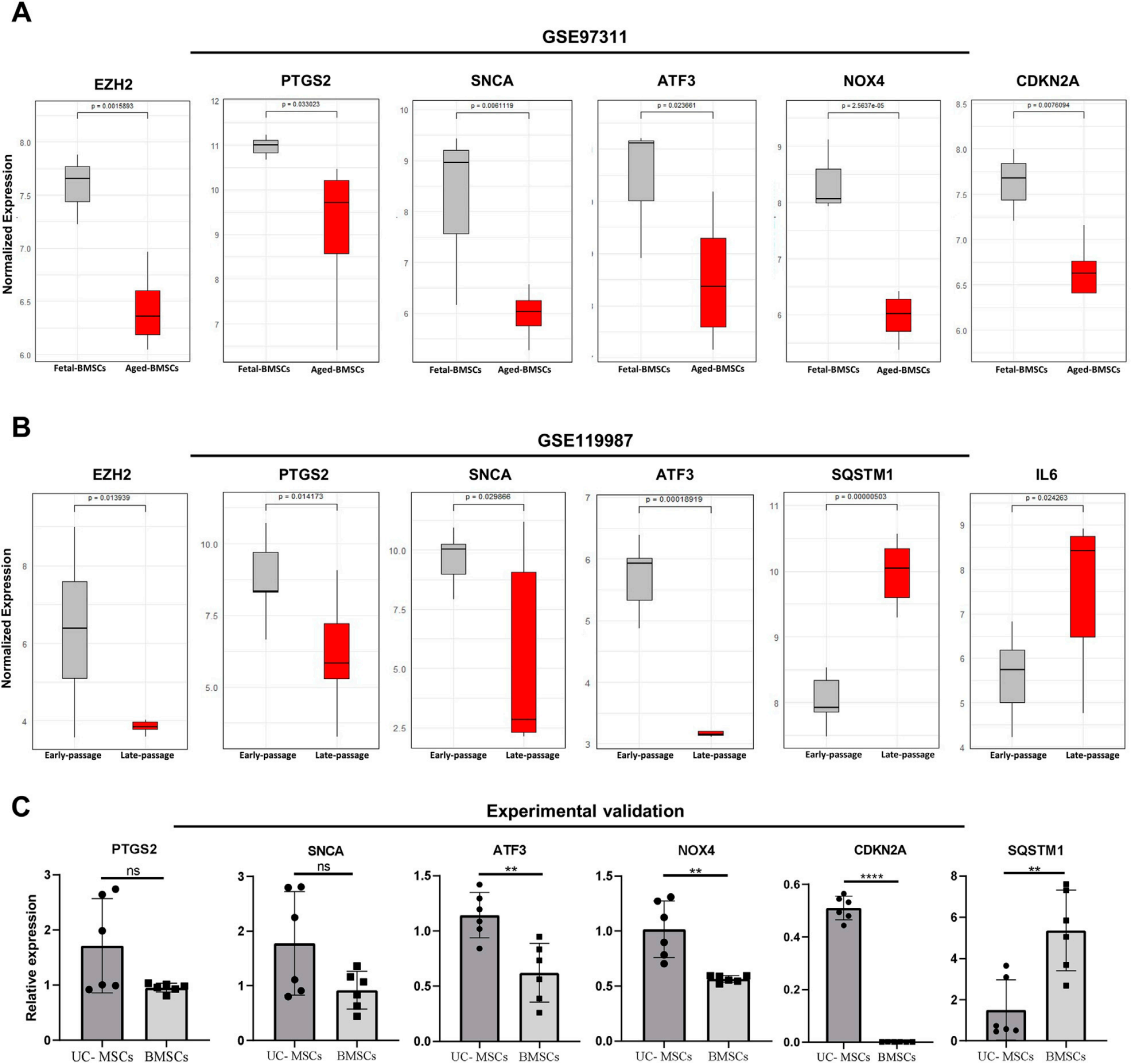
Validation of the hub genes of FRDEGs. **(A, B)** Expression of common genes between hub genes of FRDEGs and validation datasets GSE97311 and GSE119987 (EZH2, PTGS2, SNCA, ATF3), and the shared genes between hub genes of FRDEGs and each dataset (NOX4 and CDKN2A between the GSE97311 dataset and FRDEGs; SQSTM1 and IL6 between the GSE119987 dataset and FRDEGs); **(C)** Experimental validation with bar graphs comparing relative expression in young umbilical cord MSCs (UC- MSCs) and aged bone marrow MSCs (BMSCs) for PTGS2, SNCA, ATF3, NOX4, CDKN2A, and SQSTM1 genes. Statistical significance is indicated above each comparison. Data are means ± SEM, n = 6. Statistical significance was assessed using Two-tailed unpaired t-tests. *P < 0.05; **P < 0.01; ***P < 0.001; ****P < 0.0001; ns: no significant. Origin of the datasets: GSE97311, BMSCs derived from fetal (55 days post conception/7–8 weeks) and aged/adult (60 – 74 years) donors; GSE119987, human umbilical cord MSCs (UC-MSCs) from early (P2) and late (P13-P16) passages. FRDEGs: ferroptosis-related differentially expressed genes.

After analyzing the expression patterns and GSEA of DEGs in the validation datasets GSE97311 and GSE119987 (Supplementary Material 2, Supplementary Figure S1), their overlap with ferroptosis-related genes revealed that they shared 34 and 59 ferroptosis-associated genes, respectively (Supplementary Figure S2). Subsequently, the expression levels of 20 FRDEGs hub genes were analyzed across in both datasets. The results indicated that the expression of SNCA, PTGS2, EZH2, and ATF3 genes was consistent with the hub genes related to ferroptosis across both datasets, all exhibiting reduced expression in adult MSCs. Furthermore, the expression trends of NOX4 and CDKN2A in GSE97311, as well as SQSTM1 and IL6 in GSE119987, resembled those observed for the hub genes in GSE68374, showing decreased and increased expression respectively (Figures 5A,B).

### Gene expression analysis results

Based on the qRT-PCR results, the expression levels of the validated genes well reflected the patterns observed in the bioinformatic analyses (Figure 5C). This consistency between the experimental validation and the computational predictions further confirms the validity of the bioinformatic data and indicates that the identified genes are strongly associated with the processes of ferroptosis and MSCs aging.

## Discussion

Aging is an irreversible physiological process characterized by the gradual decline of bodily functions, accompanied by various molecular and cellular changes. These include mitochondrial dysfunction, DNA damage, telomere shortening, lipid peroxidation, and oxidative modifications of proteins. Such alterations not only accelerate the aging process but also serve as significant risk factors for numerous age-related diseases, highlighting the need for innovative therapeutic strategies (Zhu et al., 2021; Yang et al., 2024). Regenerative medicine, especially the application of mesenchymal stem cells (MSCs), offers promising avenues for combating aging-related frailty (Zhu et al., 2021). However, factors such as pharmacological interventions and the aging process itself can profoundly impact MSCs function in tissue repair and regeneration (Wang X. et al., 2023). To enhance therapeutic outcomes, various strategies have been explored to modulate MSCs phenotypes and improve their regenerative capacity. One potentially effective approach is the inhibition of ferroptosis, given its established role in aging (Mazhar et al., 2021; Larrick et al., 2020; Zhou et al., 2020). Understanding the relationship between ferroptosis and aging in MSCs could unveil novel biomarkers and diagnostic tools, providing new perspectives for assessing and targeting MSCs aging.

In the present bioinformatics study, all three datasets related to MSCs aging were initially subjected to GSEA. The results indicated that senescence-related genes in MSCs are involved in pathways associated with ferroptosis, including oxidative stress, lipid peroxidation, as well as fatty acid. It has been reported that disruption in iron metabolism leads to excessive accumulation of iron ions in aging cells, resulting in heightened ROS production that can lead to DNA damage, inhibition of repair function, cell cycle arrest, and subsequently accelerate the aging process (Calabrese et al., 2008). Denham-Harman was the first to suggest that ROS, which accumulates over time, is the main cause of the aging process (Goswami, 2020). In this context, research has shown that oxidative stress resulting from the accumulation of ROS significantly contributes to the skin aging process (Rinnerthaler et al., 2015). Additionally, other studies confirmed that heightened oxidative stress is a primary factor in the aging processes associated with neurological diseases (Calabrese et al., 2008; Mariani et al., 2005; Liguori et al., 2018). Several studies have demonstrated that oxidative stress is a key driver of lipid peroxidation. This process generates reactive lipid peroxides, such as malondialdehyde (MDA) and 4-hydroxy-2-nonenal, which can induce various forms of DNA damage, contribute to aging, and promote related disorders. Additionally, lipid peroxidation can further intensify oxidative stress; as the accumula tion of lipid peroxides may lead to increased ROS levels, establishing a vicious cycle that amplifies cellular damage and accelerates the aging process (Yang et al., 2024). Additionally, the GSEA results revealed that aging-related genes are involved in pathways such as PI3K/AKT/mTOR and p53. The phosphatidylinositol 3-kinase (PI3K)/AKT signaling pathway plays a critical role in regulating diverse cellular processes including survival, proliferation, and metabolism. Inhibition of the PI3K/AKT/mTOR pathway has been shown to increase susceptibility to ferroptosis by modulating lipid metabolism through sterol regulatory element-binding protein 1 (SREBP1) (Mavaddatiyan et al., 2025). The tumor suppressor TP53, commonly known as p53, is vital in preventing tumorigenesis by mechanisms such as apoptosis, cell cycle arrest, and cellular senescence. p53 has also been identified as a key regulator of ferroptosis (Mavaddatiyan et al., 2025). For instance, in the context of Parkinson’s disease, a neurodegenerative disorder characterized by the loss of dopaminergic neurons, with aging as a major risk factor, research indicates that treatment with Fer-1, a ferroptosis inhibitor, reduces p53 levels while increasing the expression of ferroptosis regulators such as SLC7A11 and GPX4, thereby mitigating cellular senescence (Li et al., 2021).

In this study, a total of 131 potential core FRDEGs were identified. To explore their roles in MSCs senescence, various bioinformatics analyses, including GO and KEGG pathway enrichment analyses, were conducted. The results showed that these genes are further involved in cellular response to reactive oxygen species, cellular senescence, kinase activity, ferroptosis, and cancer pathways. During cell division, kinases play a critical role in ensuring accurate DNA replication and equal segregation of genetic material between daughter cells (Łukasik et al., 2021). Any deregulation of the cell cycle or transcriptional processes can result in apoptosis; however, if these issues are not addressed, they may lead to various diseases, including cancer and neurodegenerative disorders (Łukasik et al., 2021). Based on this context, ferroptosis-related genes clearly promote cellular aging by affecting kinase activity and elevating ROS production, disrupting normal cell division and compromising DNA integrity. Notably, iron is crucial in various viral infections, including Human cytomegalovirus (HCMV) (Martin, 2024). Studies have shown that during HCMV infection, the expression of transferrin receptor 1 (TFR1) increases, resulting in heightened iron levels that are critical for the initial stages of the infectio. As a result, viral infections that elevate cellular iron content may trigger ferroptosis (Martin, 2024). Additionally, HCMV infection may also accelerate immune system senescence by affecting the T cell pool, contributing to unhealthy aging (Heath J and Grant M, 2020). CMV positivity in older adults is associated with frailty, disability, and increased mortality (Aiello et al., 2017). Given the above information, it is evident that FRDEGs also have a significant role in viral infections, which may ultimately contribute to aging process of MSCs. Elevated oxidative stress and increased production of ROS are linked to various cancer types by activating proto-oncogenes and inactivating tumor suppressor genes (Wang et al., 2021). ROS also act as signaling molecules that promote abnormal cell growth and metastasis through the PI3K/AKT/mTOR and MAPK pathways (Wang et al., 2021). In addition, The cellular theory of aging suggests that human aging occurs due to cellular aging, where more cells enter senescence (Kamal et al., 2020). These senescent cells release pro-inflammatory factors through the senescence-associated secretory phenotype (SASP), which can sustain their senescence and influence the tumor microenvironment, thereby promoting tumor progression (Reynolds et al., 2024). It is most likely because the ferroptosis genes are more related to various types of cancers.

The drugs associated with 131 FRDEGs were obtained, and the top 10 drugs among them were selected. Copper, resveratrol and quercetin can be mentioned among the top 10 drugs that target these FRDEGs. It has been shown that the sensitivity of ferroptosis is reduced by copper chelators, but other types of cell death such as apoptosis, necroptosis, and alkaliptosis are not inhibited (Xue et al., 2023). Copper serves as a crucial cofactor for several metalloenzymes, particularly cytochrome c oxidase (COX). Impairment of COX function can result in the excessive generation of ROS (Li F. et al., 2022). A recent study demonstrated that copper depletion induced by bathocuproinedisulfonic (BCS) significantly enhances ferroptosis in dermal papilla cells (DPCs). This BCS-mediated copper depletion triggers metabolic reprogramming, characterized by a reduction in GSH levels and the inactivation of GPX4 (Li F. et al., 2022). In this regard, several effects of resveratrol on ferroptosis and aging process have been reported. A study suggests that resveratrol has the potential to alleviate the aging phenotype of MSCs through modulation of the SIRT1 pathway (Liu et al., 2014). Resveratrol has also been shown to protect against erastin- and RSL3-induced ferroptosis by inhibiting iron-catalyzed production of hydroxyl radicals. Furthermore, the potential for osteogenic and adipogenic differentiation in MSCs is enhanced by treatment with resveratrol (Caldarelli et al., 2015). Also, quercetin has been found to be a natural food flavonoid that has anti-inflammatory and anti-ferroptosis effects in various diseases. Studies in this field have shown that quercetin reduces ferroptosis through the positive regulation of cell viability and ferroptosis antioxidant protein levels (SLC7A11 and GPX4), reduction of inflammatory cytokines and malondialdehyde (MDA) levels (Wang Y. et al., 2023).

To investigate the effect of different factors on the expression of the obtained FRDEGs, an integrated regulatory network including kinases, TFs, and miRNAs and their relationship with these genes was built. MAPK14 and CDK1 kinases showed the highest correlation with FRDEGs. MAPK14, also known as p38MAPKα, is a member of the MAP kinase family (Yang et al., 2022). Research on osteoarthritis (OA) has revealed that the expression of growth differentiation factor 15 (GDF15) may trigger cellular senescence in cartilage cells by activating MAPK14 (Weng et al., 2022). CDK1 is the sole cyclin-dependent kinase (CDK) in mammals that is crucial for cell cycle progression, facilitating the transitions from G2 to M and G1 to S, as well as progression through G1 (Wang Q. et al., 2023). Research indicates that uncontrolled cell proliferation, a hallmark of malignancy, is often driven by alterations in CDK1 activity (Wang Q. et al., 2023).

TFs regulate cell identity and function. Among the top 10 TFs, NRF2 and ATF3 are briefly described. Nrf2/NFE2L2 is vital for oxidant and redox signaling, primarily regulating glutathione production, an antioxidant that reduces oxidative stress from ROS (Song and Long, 2020). Research has shown that Nrf2 can influence intracellular free iron levels, mitochondrial function, and modulate SLC7A11, which affect the synthesis and function of GPX4. Consequently, Nrf2 is essential in the regulation of the ferroptosis process (Song and Long, 2020). Cortical bone loss is closely linked to aging and is associated with iron accumulation (Yin et al., 2024). A study employing single-cell transcriptome analysis identified ATF3 as a key inducer of ferroptosis in osteocytes (Yin et al., 2024). Increased ATF3 expression in aged osteocytes enhances iron uptake by upregulating TFR1 and blocking cystine entry via SLC7A11 (Yin et al., 2024). Notably, Inhibiting ATF3 in aged mice significantly reduced ferroptosis and mitigated cortical bone mass loss (Yin et al., 2024).

MiRNAs are key regulators in age-related diseases such as cardiovascular and neurological disorders. They are short, non-coding RNAs that modulate gene expression epigenetically by binding to 3′-untranslated regions (3′-UTRs) of target mRNAs, leading to repression of translation or mRNA degradation (Potter et al., 2021). Their activity is influenced by endogenous sponges, like certain long non-coding RNAs (lncRNAs) and circular RNAs (circRNAs), which bind miRNAs and prevent them from targeting mRNAs. This sequestration results in gene derepression, affecting cellular functions and gene networks (Rincón-Riveros et al., 2021). Numerous studies have demonstrated the pivotal role of miRNAs in MSCs aging, indicating that dysregulation—either downregulation or upregulation—of specific miRNAs within MSCs can trigger cellular senescence and contribute to the aging process (Potter et al., 2021). Based on the obtained results, the observed upregulation of certain miRNA target genes may be attributable to specific endogenous sponge mechanisms, warranting further investigation into their roles in MSCs aging. Additionally, the downregulation of certain FRDEGs, which are known to induce ferroptosis, might be influenced by complex gene regulatory networks in which modulation of other gene expressions impacts their transcription.

To identify main ferroptosis genes associated with MSCs aging, we validated the hub genes of FRDEGs using two external datasets (GSE97311, GSE119987). The findings suggest that SNCA, PTGS2, EZH2, and ATF3, which were downregulated in both late-passage UC-MSCs and BMSCs from adult donors, may play a significant role in regulating the aging process of MSCs via ferroptosis, regardless of their source. SNCA encodes the protein alpha-synuclein, which is predominantly expressed in neurons and plays a critical role in the regulation of synaptic function (Figueroa, 2020; Srinivasan et al., 2021). Recent studies have identified α-synuclein in bones and adipose tissue as well (Figueroa, 2020). Evidence indicates that SNCA is involved in regulating skeletal homeostasis; specifically, downregulation of its expression in osteoprogenitor cells leads to a mild osteogenic phenotype (Figueroa, 2020). This finding suggests that SNCA may work synergistically with bone cells and bone marrow cells to help protect against low bone mass (Figueroa, 2020). Prostaglandin-endoperoxide synthase 2 (PTGS2), commonly known as cyclooxygenase 2 (COX-2) (Anamthathmakula and Winuthayanon, 2021), serves as a rate-limiting enzyme in the production of prostaglandins (PGs) and is upregulated in response to inflammatory, tumorigenic, and hypoxic conditions (Wu et al., 2023). Studies have shown that hypoxia-induced activation of COX-2 leads to an increased release of prostaglandin E_2_ (PGE_2_) and vascular endothelial growth factor (VEGF). Conversely, inhibiting COX-2 activity results in decreased expression of VEGF, which is a key stimulator of osteogenesis (Wu et al., 2023). Endothelial cells (ECs) produce and secrete COX-2-dependent PGE2 and VEGF, which subsequently signal to BMSCs in a paracrine manner through EP1/2 and VEGFR pathways, promoting the phosphorylation of extracellular signal-regulated kinase (ERK) signaling and facilitating osteogenic differentiation (Wu et al., 2023). Enhancer of zeste homolog 2 (EZH2), a histone methyltransferase, plays a crucial role in regulating gene expression through chromatin remodeling (Li Y. et al., 2022). In a study, researchers found that the aging of mesenchymal stem/progenitor cells (MSPCs) is regulated epigenetically by Ezh2, which trimethylates histone H3 at lysine 27 (H3K27me3) (Li et al., 2017). Ezh2 exerts its effects through H3K27me3 by repressing essential genes that drive cellular senescence (Li et al., 2017). The deletion of Ezh2 in mice during early puberty led to accelerated cellular senescence, a decrease in MSPC cell populations, and impaired osteogenesis as well as osteoporosis (Li et al., 2017).

To definitively confirm the similarity in expression patterns of FRDEGs in MSCs from different sources, we performed a rigorous comparative analysis of their expression profiles using qRT-PCR validation. Our objective was to compare two fully aged *versus* fully young cellular states, irrespective of cell source or varying aging conditions, in order to assess whether qRT-PCR results are concordant with bioinformatic predictions. Specifically, to ensure the aged and young statuses of the cells, we analyzed MSCs derived from bone marrow of an aged donor (70 years) at passage 16 and MSCs derived from umbilical cord from a young donor at early passage (P3). This design captures extremes of biological age (old vs young) and tests whether the gene expression patterns identified in the initial datasets (GSE68374, GSE97311, and GSE119987) are recapitulated under *in vitro* conditions. Notably, the qRT-PCR results were concordant with the bioinformatic predictions and provide compelling evidence that these FRDEGs may play meaningful roles in the molecular pathways underpinning aging of MSCs, irrespective of their source. This validation highlights the potential of these genes as cellular aging biomarkers and underscores their promise as therapeutic targets for interventions aimed at mitigating age-related functional decline in mesenchymal stem cells.

## Conclusion

This study used bioinformatic analysis to investigate the role of ferroptosis in aging MSCs by comparing gene expression in fetal and adult MSCs. Our findings indicated that ferroptosis-related genes are involved in multiple pathways and biological functions related to cell fate and aging. Changes in the expression of these ferroptosis genes may affect MSCs activity by modulating several signaling pathways, including the prominent SIRT1, Nrf2, and Wnt/β-catenin pathway, as well as secondary pathways such as EP1/2 and VEGFR pathways. In addition, promising therapeutic drugs that can target these pathways, such as copper, resveratrol, and quercetin were discovered, suggesting new pathways to prevent aging-related degeneration in MSCs. This study reveals the complexity of aging of MSCs and the critical function of ferroptosis, providing new avenues for therapeutic intervention and laying the groundwork for future research on aging and related diseases.

## Data Availability

The GSE68374, GSE97311, and GSE119987 datasets were obtained from GEO database (https://www.ncbi.nlm.nih.gov/geo/). Additionally, ferroptosis-related genes, including drivers, suppressors, markers, and unclassified genes, were sourced from the online database FerrDb (http://www.zhounan.org/ferrdb/current/). Other data that support the findings of this study are available upon request to the corresponding author.
