# Supplementary material for: Exploring the involvement of ferroptosis-associated genes and pathways in mesenchymal stem cell aging through bioinformatics analysis
Source: Front Aging. 2025 Oct 15;6:1509267. doi: 10.3389/fragi.2025.1509267 (PMC12568457; doi:10.3389/fragi.2025.1509267)
Supplement: Supplementary file 2 [file Table1.docx]

**Supplementary Table 1.** Primers for RT-PCR analysis

| **Primer names** | **Primer sequences** |
| --- | --- |
| PTGS2 | Forward: TCAGCCATACAGCAAATCCT  Reverse: CTTGAAGTGGGTAAGTATGTAGTG |
| **SNCA** | Forward: AAGAGGGTGTTCTCTATGTAGGC  Reverse: GCTCCTCCAACATTTGTCACTT |
| **ATF3** | Forward: CCTCTGCGCTGGAATCAGTC  Reverse: TTCTTTCTCGTCGCCTCTTTTT |
| **NOX4** | Forward: TGACGTTGCATGTTTCAGGAG  Reverse: AGCTGGTTCGGTTAAGACTGAT |
| **CDKN2A** | Forward: ATGGAGCCTTCGGCTGACT  Reverse: GTAACTATTCGGTGCGTTGGG |
| **SQSTM1** | Forward: GACTACGACTTGTGTAGCGTC  Reverse: AGTGTCCGTGTTTCACCTTCC |
| **GAPDH** | Forward: CAG GTG GTC TCC TCT GAC TTC AAC  Reverse: AGG GTC TCT CTC TTC CTC TTG TGC |

**Supplementary Table 2.** Main miRNAs targeting FRDEGs

| **miRNAs**  **(mirDIP ∩ miRWalk ∩ ENCORI)** | **Gene Symbol** | **Gene Expression** | **Ferroptosis Driver / Suppressor/** **Marker/** **Unclassified** |
| --- | --- | --- | --- |
| hsa-miR-324-5p  hsa-miR-4640-5p  hsa-miR-4726-5p | ATF3 | Down regulation | Driver/ Unclassified |
| hsa-miR-129-1-3p  hsa-miR-4664-3p | CAMKK2 | Up regulation | Suppressor |
| hsa-miR-2682-5p  hsa-miR-506-5p  hsa-miR-510-5p  hsa-miR-552-3p | CAV1 | Down regulation | Suppressor |
| hsa-miR-15b-5p  hsa-miR-16-5p  hsa-miR-195-5p  hsa-miR-301b-3p  hsa-miR-4524a-5p  hsa-miR-4524b-5p  hsa-miR-503-5p | CCDC6 | Down regulation | Driver |
| hsa-miR-1296-5p  hsa-miR-3126-5p  hsa-miR-4739  hsa-miR-4756-5p | CD44 | Down regulation | Suppressor |
| hsa-miR-122-5p  hsa-miR-497-5p | CDC25A | Down regulation | Suppressor |
| hsa-miR-3118  hsa-miR-7-5p | EGFR | Up regulation | Driver |
| hsa-miR-31-5p  hsa-miR-520a-5p  hsa-miR-525-5p | ELAVL1 | Down regulation | Driver/ Unclassified |
| hsa-miR-5047 | EZH2 | Down regulation | Suppressor |
| hsa-miR-1224-5p | FADS1 | Down regulation | Driver |
| hsa-miR-143-3p  hsa-miR-3144-3p  hsa-miR-363-3p  hsa-miR-425-5p  hsa-miR-506-3p  hsa-miR-514b-5p | FAR1 | Down regulation | Driver |
| hsa-miR-3622a-5p  hsa-miR-483-3p  hsa-miR-654-5p | FURIN | Down regulation | Suppressor |
| hsa-miR-4731-5p | FXN | Down regulation | Suppressor |
| hsa-miR-1323 | GCLC | Down regulation | Suppressor |
| hsa-miR-1911-5p | GJA1 | Up regulation | Driver |
| hsa-miR-574-3p | IL6 | Up regulation | Driver |
| hsa-miR-1343-3p  hsa-miR-338-3p  hsa-miR-574-5p | IREB2 | Down regulation | Driver/ Unclassified |
| hsa-miR-1271-5p  hsa-miR-143-3p  hsa-miR-181d-5p  hsa-miR-202-5p  hsa-miR-3164  hsa-miR-330-5p  hsa-miR-6088  hsa-miR-6509-3p  hsa-miR-6509-5p  hsa-miR-877-5p  hsa-miR-888-5p | KRAS | Up regulation | Driver |
| hsa-miR-28-5p  hsa-miR-3163  hsa-miR-513b-5p  hsa-miR-668-3p  hsa-miR-708-5p  hsa-miR-760 | LIFR | Down regulation | Driver |
| hsa-miR-105-5p | LPIN1 | Down regulation | Driver |
| hsa-miR-129-1-3p  hsa-miR-1321  hsa-miR-4701-5p  hsa-miR-4756-5p  hsa-miR-514a-5p  hsa-miR-654-3p  hsa-miR-769-5p | MAFG | Up regulation | Unclassified |
| hsa-miR-2278  hsa-miR-489-3p  hsa-miR-513a-5p | MAPK14 | Up regulation | Driver/ Unclassified |
| hsa-miR-130b-3p  hsa-miR-216b-5p  hsa-miR-339-5p  hsa-miR-4640-5p  hsa-miR-4701-5p  hsa-miR-4712-5p | MDM4 | Down regulation | Driver |
| hsa-let-7c-5p  hsa-let-7d-5p  hsa-let-7f-5p  hsa-miR-124-3p  hsa-miR-186-5p  hsa-miR-3622b-5p  hsa-miR-4500  hsa-miR-766-5p | MIB1 | Down regulation | Driver |
| hsa-miR-500a-3p  hsa-miR-513c-5p  hsa-miR-758-3p | NCOA3 | Down regulation | Suppressor |
| hsa-miR-3196 | NDRG1 | Up regulation | Driver |
| hsa-miR-105-5p  hsa-miR-10a-5p  hsa-miR-10b-5p  hsa-miR-18a-5p  hsa-miR-18b-5p  hsa-miR-27a-3p  hsa-miR-30e-5p  hsa-miR-3129-5p  hsa-miR-449c-5p | NEDD4 | Down regulation | Suppressor |
| hsa-miR-105-5p  hsa-miR-106a-5p  hsa-miR-17-5p  hsa-miR-20a-5p  hsa-miR-20b-5p  hsa-miR-2682-5p  hsa-miR-449c-5p  hsa-miR-519d-3p  hsa-miR-664b-3p  hsa-miR-875-5p  hsa-miR-93-5p | NEDD4L | Down regulation | Suppressor |
| hsa-miR-146a-5p  hsa-miR-378g  hsa-miR-3918  hsa-miR-489-3p  hsa-miR-510-5p  hsa-miR-625-5p | NF2 | Down regulation | Suppressor |
| hsa-miR-4661-5p | NFE2L2 | Up regulation | Suppressor/ Marker/ Unclassified |
| hsa-let-7d-5p  hsa-miR-4458  hsa-miR-515-5p  hsa-miR-942-5p | NRAS | Down regulation | Driver |
| hsa-miR-107  hsa-miR-497-5p | PDK4 | Up regulation | Suppressor |
| hsa-miR-1247-5p  hsa-miR-2355-5p  hsa-miR-329-3p  hsa-miR-362-3p  hsa-miR-483-3p  hsa-miR-485-5p  hsa-miR-5691 | PHF21A | Down regulation | Driver |
| hsa-miR-3184-5p  hsa-miR-423-5p | PML | Down regulation | Suppressor |
| hsa-miR-149-5p | PRDX6 | Down regulation | Suppressor |
| hsa-miR-154-5p  hsa-miR-183-5p  hsa-miR-362-5p  hsa-miR-873-5p | PRKCA | Down regulation | Driver |
| hsa-miR-543 | PTGS2 | Down regulation | Marker |
| hsa-let-7d-5p  hsa-let-7f-5p | RRM2 | Down regulation | Suppressor/ Unclassified |
| hsa-let-7c-5p  hsa-let-7f-5p  hsa-miR-1185-5p  hsa-miR-181b-5p  hsa-miR-200b-3p  hsa-miR-2681-5p  hsa-miR-3129-5p  hsa-miR-432-5p  hsa-miR-98-5p | SCD | Down regulation | Suppressor |
| hsa-miR-934 | SLC16A1 | Down regulation | Suppressor |
| hsa-miR-1286  hsa-miR-138-5p  hsa-miR-146a-5p  hsa-miR-210-3p  hsa-miR-296-3p  hsa-miR-3126-5p  hsa-miR-3129-5p  hsa-miR-378a-3p  hsa-miR-378b  hsa-miR-378d  hsa-miR-378e  hsa-miR-378f  hsa-miR-378h  hsa-miR-378i  hsa-miR-483-5p  hsa-miR-505-3p  hsa-miR-508-3p  hsa-miR-518d-5p  hsa-miR-519b-5p  hsa-miR-519c-5p  hsa-miR-520c-5p  hsa-miR-758-3p | SLC38A1 | Down regulation | Driver |
| hsa-miR-20a-5p  hsa-miR-20b-5p  hsa-miR-574-5p | SLC40A1 | Up regulation | Suppressor |
| hsa-miR-487a-3p  hsa-miR-519c-3p  hsa-miR-5579-3p | SMAD7 | Up regulation | Driver |
| hsa-miR-3614-5p | SNCA | Down regulation | Driver |
| hsa-miR-1914-3p  hsa-miR-5194 | SQSTM1 | Up regulation | Suppressor |
| hsa-miR-532-3p | SREBF2 | Down regulation | Suppressor |
| hsa-miR-193a-3p | STMN1 | Down regulation | Unclassified |
| hsa-miR-125a-5p  hsa-miR-455-3p  hsa-miR-744-5p | SUV39H1 | Down regulation | Suppressor |
| hsa-miR-132-5p  hsa-miR-199b-3p  hsa-miR-769-5p | TFAM | Down regulation | Suppressor |
| hsa-miR-448 | TLR4 | Up regulation | Driver |
| hsa-miR-4319  hsa-miR-5194 | TNFAIP3 | Up regulation | Driver |
| hsa-let-7e-5p  hsa-miR-1294  hsa-miR-150-5p | TP53 | Down regulation | Driver |
| hsa-miR-187-3p  hsa-miR-29a-3p  hsa-miR-99b-5p | TRIB2 | Down regulation | Suppressor |
| hsa-miR-1271-5p | TRIB3 | Up regulation | Unclassified |
| hsa-miR-150-5p  hsa-miR-760  hsa-miR-96-5p | TSC22D3 | Up regulation | Unclassified |
| hsa-miR-302a-3p  hsa-miR-3179  hsa-miR-520d-3p  hsa-miR-588  hsa-miR-627-5p | TXNIP | Up regulation | Unclassified |
| hsa-miR-205-5p  hsa-miR-491-5p  hsa-miR-873-3p | TXNRD1 | Up regulation | Unclassified |
| hsa-miR-302a-3p  hsa-miR-373-3p  hsa-miR-519d-3p  hsa-miR-526b-3p | VLDLR | Up regulation | Unclassified |
| hsa-miR-139-5p  hsa-miR-199a-3p  hsa-miR-199b-3p  hsa-miR-200c-3p  hsa-miR-431-5p  hsa-miR-642a-3p  hsa-miR-642b-3p | ZEB1 | Down regulation | Driver |
