# Supplementary material for: Exploring the involvement of ferroptosis-associated genes and pathways in mesenchymal stem cell aging through bioinformatics analysis
Source: Front Aging. 2025 Oct 15;6:1509267. doi: 10.3389/fragi.2025.1509267 (PMC12568457; doi:10.3389/fragi.2025.1509267)
Supplement: Supplementary file 3 [file Table2.docx]

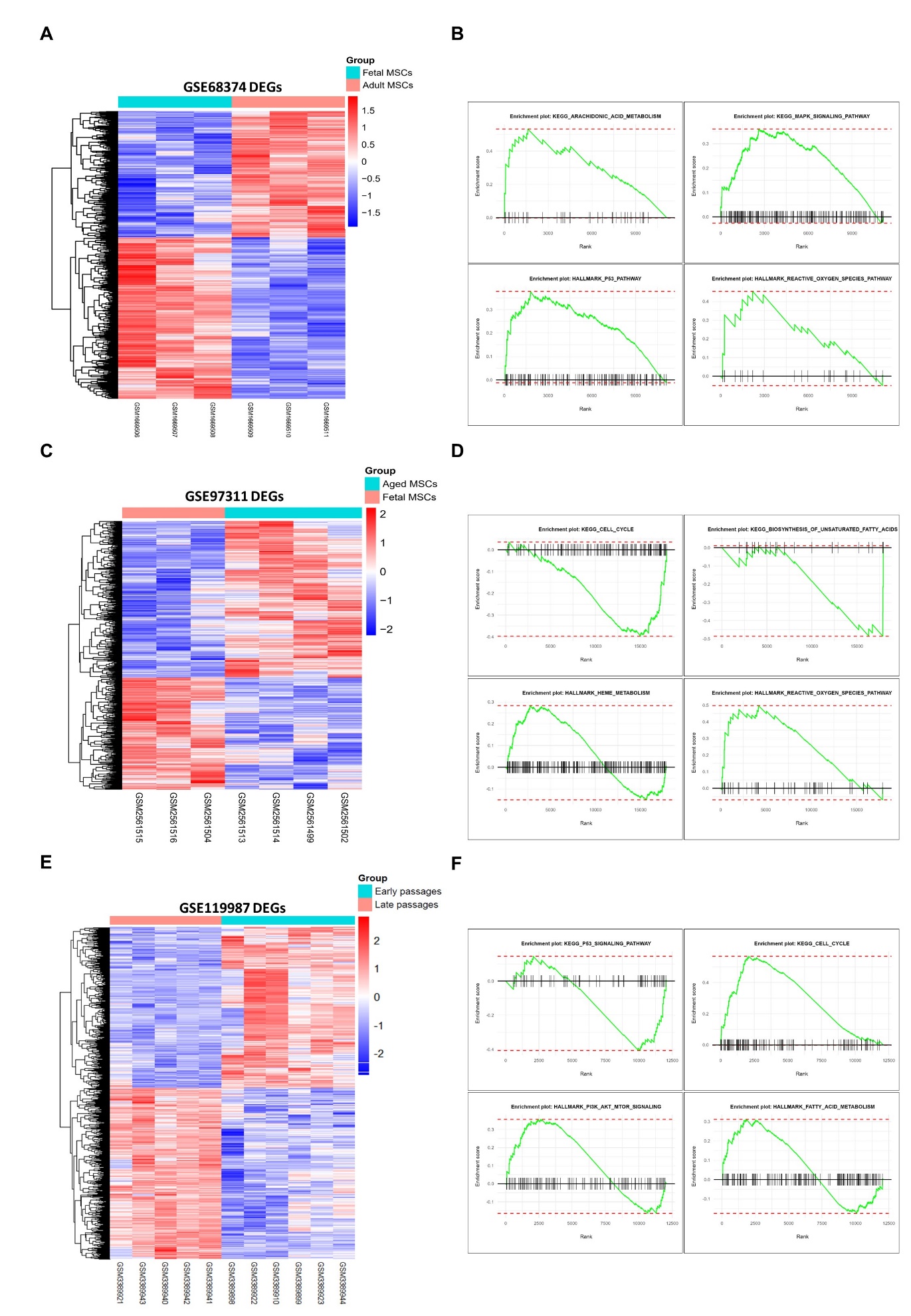


**Supplementary Fig.1.** Analysis of DEGs expression patterns and gene set enrichment analysis (GSEA) for the datasets. (A-B) Heatmap and GSEA for GSE68374; (C-D) Heatmap and GSEA for GSE97311; (E-F) Heatmap and GSEA for GSE119987.


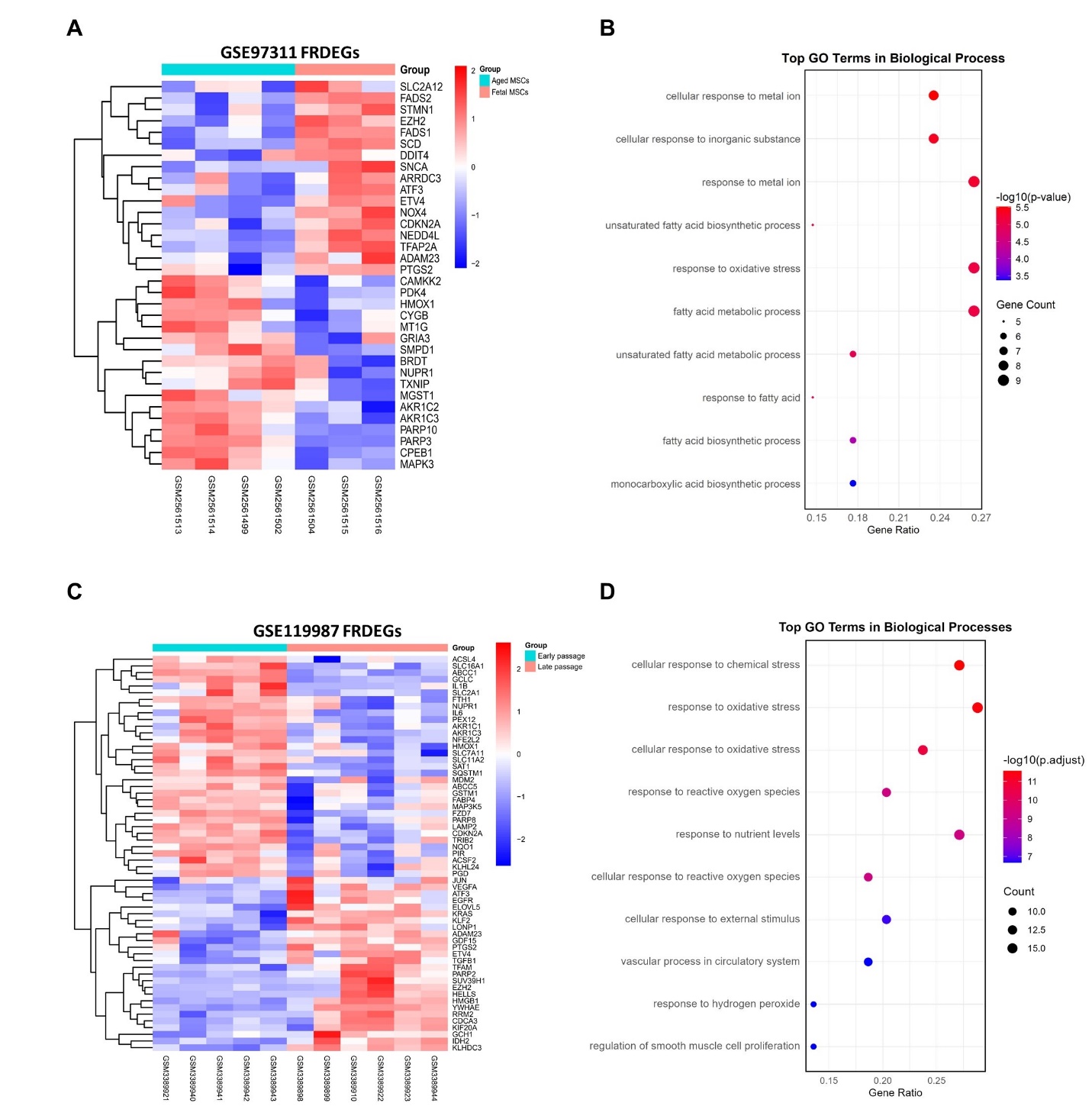


**Supplementary Fig.2.** Evaluation of FRDEGs' expression patterns and biological functions in validation datasets. (A-B) Heatmap and functional analysis of GSE97311; (C-D) heatmap and functional analysis of GSE119987.
